# A Real-Time PCR Assay for Detection of Low *Pneumocystis jirovecii* Levels

**DOI:** 10.3389/fmicb.2021.787554

**Published:** 2022-01-11

**Authors:** Susana Ruiz-Ruiz, Carolina A. Ponce, Nicole Pesantes, Rebeca Bustamante, Gianna Gatti, Viviana San Martin, Mireya Gutierrez, Pamela Bórquez, Sergio L. Vargas, Fabien Magne, Enrique J. Calderón, Vicente Pérez-Brocal, Andrés Moya

**Affiliations:** ^1^Fundación para el Fomento de la Investigación Sanitaria y Biomédica de la Comunitat Valenciana (FISABIO)-Salud Pública, València, Spain; ^2^Centro de Investigación Biomédica en Red en Epidemiología y Salud Pública (CIBEResp), Instituto de Salud Carlos III, Madrid, Spain; ^3^Programa de Microbiología y Micología, Facultad de Medicina, Instituto de Ciencias Biomédicas, Universidad de Chile, Santiago, Chile; ^4^Servicio Médico Legal, Santiago, Chile; ^5^Hospital Universitario Virgen del Rocío, Instituto de Biomedicina de Sevilla, Consejo Superior de Investigaciones Científicas (CSIC), and Universidad de Sevilla, Seville, Spain; ^6^Instituto de Biología Integrativa de Sistemas (I2Sysbio), Universitat de València and Consejo Superior de Investigaciones Científicas (CSIC), València, Spain

**Keywords:** real-time PCR, low *P. jirovecii* loads, major surface glycoproteins, mitochondrial large subunit rRNA, simultaneous detection

## Abstract

Here we report a new real-time PCR assay using SYBR Green which provides higher sensitivity for the specific detection of low levels of *Pneumocystis jirovecii*. To do so, two primer sets were designed, targeting the family of genes that code for the most abundant surface protein of *Pneumocystis* spp., namely the major surface glycoproteins (Msg), and the mitochondrial large subunit rRNA (mtLSUrRNA) multicopy gene, simultaneously detecting two regions. PCR methods are instrumental in detecting these low levels; however, current nested-PCR methods are time-consuming and complex. To validate our new real-time Msg-A/mtLSUrRNA PCR protocol, we compared it with nested-PCR based on the detection of *Pneumocystis* mitochondrial large subunit rRNA (mtLSUrRNA), one of the main targets used to detect this pathogen. All samples identified as positive by the nested-PCR method were found positive using our new real-time PCR protocol, which also detected *P. jirovecii* in three nasal aspirate samples that were negative for both rounds of nested-PCR. Furthermore, we read both rounds of the nested-PCR results for comparison and found that some samples with no PCR amplification, or with a feeble band in the first round, correlated with higher *Ct* values in our real-time Msg-A/mtLSUrRNA PCR. This finding demonstrates the ability of this new single-round protocol to detect low *Pneumocystis* levels. This new assay provides a valuable alternative for *P. jirovecii* detection, as it is both rapid and sensitive.

## Introduction

*Pneumocystis jirovecii* is an opportunistic fungal pathogen. Children represent one of the main reservoirs in humans but predominantly suffer subclinical infection; therefore, *P. jirovecii* detection is hindered given the absence of overt signs or symptoms (Morris et al., 2008). In this context, PCR methods are instrumental in detecting low *P. jirovecii* levels, as colonization is characterized by a low fungal burden in the respiratory tract, hampering its detection. Several PCR and real-time PCR assays have been standardized targeting different single-copy genes such as superoxide dismutase (*sod*) (Morilla et al., 2019), dihydrofolate reductase (*dhfr*) (Alanio et al., 2016), cytochrome c oxidase (*cox-1*), or the kexin-like serine protease (*kex1*) gene (Dunaiski et al., 2018). Increased detection sensitivity has also been reported using multicopy genes, such as the mitochondrial large subunit ribosomal RNA (*mtLSUrRNA*) (Morris et al., 2008; Alanio et al., 2011; Fraczek et al., 2019; Chotiprasitsakul et al., 2020; Yang et al., 2020), the mitochondrial small subunit ribosomal RNA (*mtSSUrRNA*) (Dellière et al., 2019) or the major surface glycoprotein (*Msg*) gene family (Rudramurthy et al., 2018; Bossart et al., 2020; Gits-Muselli et al., 2020). In addition, multiplex real-time PCR methods have also been developed where more than one gene is detected to characterize *P. jirovecii* specific single-nucleotide polymorphisms located at different loci (Esteves et al., 2011; Montesinos et al., 2017). However, to date, the nested-PCR targeting the *Pneumocystis* mitochondrial large subunit rRNA (*mtLSUrRNA*) remains one of the most widely used techniques for detecting *P. jirovecii* at low levels. Nested-PCR consists of two sequential rounds of conventional PCR, in which the second round of PCR amplifies an internal region of the amplicons generated in the first round (Wakefield et al., 1990; Wakefield, 1996). The two rounds of PCR make this technique highly sensitive, enabling the detection of low fungal burdens (Ponce et al., 2010; Özkoç et al., 2016). This nested-PCR is the method of choice for detecting low *P. jirovecii* loads in non-invasive samples such as oral lavage, nasopharyngeal aspirates, or in lung samples. However, this method is laborious, time-consuming, and entails considerable risk of contamination, increasing the risk of detecting false positives (Chabé et al., 2014). By contrast, real-time PCR has several advantages, including relatively rapid assay times (hours), reliability, and ease of replicating analyses.

Previous studies have attempted to use the burden of *P. jirovecii* to differentiate between acceptable levels of colonization and those where *P. jirovecii* pneumonia (PcP) represents a health risk (Rudramurthy et al., 2018). However, even if standardized underlying causes of immunosuppression are met, the *Pneumocystis* burden in itself is not specific enough to define PcP. Indeed, there is a gray zone with burden overlapping where PcP and colonization cases coexist (Robert-Gangneux et al., 2014; Fauchier et al., 2016; Sarasombath et al., 2021). Therefore, more precise information on the colonization status will be provided by improving specificity for the diagnosis of PcP vs. colonization in lower respiratory tract samples like bronchoalveolar lavage (BAL), endotracheal aspirate (ETA), and in less invasive samples like sputum or nasopharyngeal aspirates (NA) and oral lavage. Such improvements will enable the identification of pathogenic effects and diagnosis of emerging *Pneumocystis* pneumonia in the non-HIV-related population (Robert-Gangneux et al., 2014; Fauchier et al., 2016; Sarasombath et al., 2021) and the non-invasive diagnosis of *Pneumocystis* in newborns and young infants.

Recently, the complete genome assemblies for *Pneumocystis* species infecting humans (*P. jirovecii*), rats (*P. carinii*), and mice (*P. murina*) have been reported and are available from the NCBI Umbrella project PRJNA223519 (Ma et al., 2016). The most significantly enriched domains in *P. jirovecii* and *P. carinii* are the multicopy gene families encoding the major surface glycoproteins (Msg), which are absent in another fungal species analyzed by Ma et al. (2016). In the aforementioned study based on sequence similarity, Msg proteins were classified into five families: Msg-A, -B, -C, -D, and –E (Ma et al., 2016), where Msg-A proved to be by far the largest family. Our study aims to develop and validate a more sensitive new real-time single-round PCR method based on simultaneously amplifying two regions, the mtLSUrRNA and the Msg-A multicopy gene family, a nested-PCR alternative for low levels of *Pneumocystis* diagnosis. We aim to increase specificity by enhancing the recognition of *P. jirovecii*-specific targets and reducing false-positive results caused by contamination or cross-reactions.

## Materials and Methods

### Clinical Samples and DNA Extraction Protocol

This study considered 73 biological samples, including 60 lung autopsies of healthy infants who died due to sudden death (LUNG 1–54, LUNG 1650, 1783, 895, 2604, 73B, and 65B), seven nasal aspirates (NA 102, 113, 119, 143, 150, 501, 502), three BAL (BAL FQ773, 953 and C965) and three gargles (G 05v4, 04v5, and 02v5). Diagnosis of autopsy samples from infants who suffered sudden unexpected death was established based on clinical history, macroscopic examination and dissection with histological sampling of major organs and laboratory tests including toxicology determinations. Postmortem bacterial cultures were not considered and detection of positive cases of respiratory viruses were excluded. Samples were categorized as *P. jirovecii*-positive based on the presence of *P. jirovecii* specific DNA by nested-PCR and, in the case of lung samples, also by the immunofluorescence-based detection of ascus forms with MERIFLUOR *Pneumocystis* (Meridian Bioscience).

The Ethics Commission for Studies in Human Subjects of the University of Chile School of Medicine approved this study under protocol CEISH #092-2013. All methods were performed following the principles of the Declaration of Helsinki for medical research involving human subjects and following the relevant local guidelines and regulations.

Total DNA was extracted and purified following method B described by Ruiz-Ruiz et al. (2019). Said method consisted of DNA extraction with the QIAamp DNA Mini kit (Qiagen) and previous additional steps, including pre-treatment (samples were homogenized by magnetic stirrer agitation in sterile PBS), bead-beating and Phenol: Chloroform: Isoamyl alcohol steps. All manipulations were made inside a biosafety cabinet using new sterile equipment. DNA extraction was carried out in groups of five samples and each group with its respective extraction controls. In the case of lung tissue samples, it is well-known that *Pneumocystis* distribution in the competent host lung is focal in lung samples. However, previous studies showed that sampling 3% of the weight of the right upper lobe is enough to obtain excellent sensitivity in order to identify low levels of *Pneumocystis* (Vargas et al., 2017). DNA concentrations of all samples were measured using the Qubit double-stranded DNA (dsDNA) BR assay kit (Invitrogen) on a Qubit 4.0 Fluorometer (Invitrogen).

### mtLSUrRNA Nested-PCR

*Pneumocystis* DNA was detected first using the nested-PCR procedure, amplifying part of the gene coding for mitochondrial large subunit ribosomal RNA (mtLSUrRNA) (Wakefield et al., 1990). The first round of nested-PCR was performed using the oligonucleotide primers pAZ102-E and pAZ102-H, utilizing 5 μL of DNA preparation. Amplification was performed under the following conditions: 94°C for 5 min, 94°C for 1.0 min, 55°C for 1.0 min, and 72°C for 2.0 min for 40 cycles. The second round of nested-PCR was performed using pAZ102-X and pAZ102-Y primers, which are internal to the first set of primers and specific for *P. jirovecii* taking 1 μL from the first-round PCR product under the following PCR conditions: 94°C for 5 min, 94°C for 1.0 min, 56°C for 1.0 min, and 72°C for 2.0 min for 10 cycles and 94°C for 1.0 min, 64°C for 1.0 min and 72°C for 2.0 min for 30 cycles. Samples were categorized as follows: (a) *Pneumocystis*-positive, when the *P. jirovecii* DNA specific 267 bp DNA band was obtained by nested-PCR in the sample, or one or more analyzed lobes in the case of lung samples, and (b) *Pneumocystis*-negative if no *P. jirovecii* DNA was documented. *Pneumocystis*-negative samples were analyzed twice, starting from the tissue or fluid. Controls were run simultaneously with samples. Positive controls were lung tissue DNA from patients with PCP. Negative controls such as water and DNA from *Pneumocystis-*negative lung tissue were used as templates in the first round PCR to control contamination, which was used *de novo* as a template for the second round PCR (negative controls). DNA amplification was performed twice for each sample. The PCR product was analyzed by electrophoresis in a 2% agarose gel and visualized with GelRed^®^ (Biotium).

### Primer Design for Real-Time Msg-A/mtLSUrRNA PCR

The two pairs of primers (Msg-AF-Msg-AR and MlsF-MlsR) were designed based on the conserved sequences in *P. jirovecii* Msg-A and mtLSUrRNA genes using the Primer Express software (Applied Biosystems) and a multiple alignment of all Msg-A DNA sequences obtained from the original publication by Ma et al. (2016) (accession code in the NCBI BioProject database PRJNA223510) or a representative number of all the complete sequences of the *P. jirovecii* mtLSUrRNA gene deposited in the NCBI and GenBank databases, respectively. These multiple alignments were obtained with the ClustalW program (Thompson et al., 2002). For the Msg alignment, the primer set contained 179 genes that were annotated in the *P. jirovecii* genome and represented all the existing variation of paralogs in a genome. The nucleotide sequence of the primer set Msg-AF and Msg-AR contained three degenerate positions each (Supplementary Figure 1). The target specificity of the new pairs of primers was evaluated with total DNA from culture of other lung pathogens such as *Aspergillus fumigatus*, *Candida albicans*, or *Cryptococcus neoformans*, which are involved in other lung infections.

### Real-Time Msg-A/mtLSUrRNA PCR Conditions

Real-time PCR was performed in a LightCycler^®^ 480 System (Roche Molecular Diagnostics) using 10 μL of a reaction mix that contained 5 μL of LightCycler^®^ FastStart DNA Master Plus SYBR Green I (Roche Diagnostics), 1.8 μL of DNase-free water, 0.3 μM of each primer and 2 μL of total DNA extraction (10 ng DNA/μL). Control samples in each run included total DNA from lung tissue *Pneumocystis-*negative, water instead of total DNA extract. Each sample was analyzed in duplicate in each independent real-time PCR assay. All the DNA extractions were adjusted to approximately 10 ng/μL and then measured with the Qubit 4 Fluorometer (Invitrogen) to allow uniformity in sample collection. Cycling conditions included incubation at 95°C for 10 min and 45 cycles of 95°C for 10 s, 58°C for 10 s, and 72°C for 15 s (when we took fluorescence measurements). The melting curves analysis was performed with the LightCycler platform software, displaying the first derivative of the fluorescence intensity vs. the temperature. Synthesis of DNA products of the expected size was confirmed by melting curve analysis and electrophoretic separation in a 2% agarose gel and staining with GelRed^®^ (Biotium). The LightCycler software plotted the fluorescence intensity against the number of cycles and provided the threshold cycle (*Ct*) value using the automatic method for each run. Sensitivity, specificity, positive predictive value (PPV), and negative predictive value (NPV) were reported only for the 60 lung autopsies of healthy infants who died due to sudden death (LUNG 1–54, LUNG 1650, 1783, 895, 2604, 73B and 65B) considering the nested-PCR as the reference method.

### Real-Time PCR Protocol From Rudramurthy’s Study

We compared the new real-time PCR protocol with the protocol developed by Rudramurthy et al. (2018), comprising a real-time quantitative PCR using Msg genes [primer pairs described by Linssen et al. (2006), F 5’-CAA AAA TAA CAY TSA CAT CAA CRA GG-3’ and R 5’-AAA TCA TGA ACG AAA TAA CCATTG C-3’]. For this comparative study, all *Pneumocystis*-positive samples were included. Because our new protocol simultaneously amplifies two regions, in addition to comparing Rudramurthy’s protocol with our Msg-A/mtLSUrRNA real-time PCR, we have also compared them with the primer set Msg-A in only one group of samples (LUNG 19–54). The real-time PCRs were performed in a LightCycler^®^ 480 System (Roche Molecular Diagnostics) using 10 μL of a reaction mix that contained 5 μL of LightCycler^®^ FastStart DNA Master Plus SYBR Green I (Roche Diagnostics), 1.8 μL of DNase-free water (in the case of using both pairs of primers) or 2.4 μL of DNase-free water (using only the primers that amplify Msg-A), 0.3 μM of each primer and 2 μL of total DNA extraction (10 ng DNA/μL). Cycling conditions were performed according to Rudramurthy et al. (2018). Control samples in each run included total DNA from lung tissue *Pneumocystis*-negative and water instead of total DNA extract. Each sample was analyzed in duplicate. Statistical comparisons between the two real-time PCR methods were made using paired *t*-tests.

## Results

### Real-Time Msg-A/mtLSUrRNA PCR Optimization

The primer sets were designed based on multiple alignments. The nucleotide sequence of the primer set Msg-AF- Msg-AR contained three degenerate positions each to amplify many related genes of the Msg-A family (Supplementary Figure 1).

For the PCR cycling conditions, we selected the program providing the most sensitive detection. Although low DNA concentration was observed in some samples, we noted that better results were obtained using diluted DNA extractions adjusted to 10 ng/μL than non-diluted DNA extraction ranging from 2118 to 41.3 ng/μL. In fact, for some samples (LUNG 19, LUNG 21, LUNG 26, and LUNG 46), no exponential amplification was observed with the undiluted DNA extraction (Supplementary Figure 2). Generally, for the LightCycler^®^ 480 System, Roche recommends the use of DNA concentrations ranging from 5 to 30 ng for real-time PCR.

The specificity of Msg-A/mtLSUrRNA PCR amplification was confirmed by melting curves and amplified products in all *P. jirovecii*-positive samples. It is worth mentioning that each pair of primers amplified a region of similar size and had similar annealing temperatures. The sufficiently different percentage of GC nucleotides (51% GC for the Msg-A region and 36% for the mtLSUrRNA region) was also important in selecting the region to design the primers, allowing the visualization of two separate melting curves according to their different *Tm* values. The results indicated whether one or two regions were amplified. The first derivative of the melting curve in the new Msg-A/mtLSUrRN real-time PCR showed two peaks with melting temperatures ranging from 81.00 to 83.00^°^C (Msg-A) and from 74.00 to 75.00°C (mtLSUrRNA), with the exception of all three nasal aspirate samples (NA 501, 502, and 113), in which only the melting curve of the Msg-A gene family was observed. Meanwhile, a single band with the expected size (around 117 bp) was observed for all the *Pneumocystis*-positive samples.

We did not observe any amplification product or melting curve profile when the primers were evaluated using other pathogens such as *Aspergillus fumigatus*, *Candida albicans*, and *Cryptococcus neoformans*, occasionally encountered in the lung.

### Comparison of the Nested-PCR and the Msg-A/mtLSUrRNA PCR

To validate our new real-time Msg-A/mtLSUrRNA PCR protocol, we compared it with the results obtained by the nested-PCR (mtLSUrRNA). Lung samples from LUNG 19 to 54, LUNG 1650, 1783, 895, 2604, 73B and 65B, nasal aspirates NA 119, 143 and 150, BAL FQ773, 953 and C965 and gargles G 05v4, 04v5 and 02v5 were categorized as *P. jirovecii*-positive given the presence of *P. jirovecii* specific DNA by nested-PCR (54/73 samples) and ascus forms by immunofluorescence (only in lung samples) (Table 1). All samples identified as *P. jirovecii-*positive using the nested-PCR method were also classified as *Pneumocystis*-positive using the real-time Msg-A/mtLSUrRNA PCR protocol with *Ct* values ranging from 25.30 to 40.00 (Table 1). However, some positive samples presented low *P. jirovecii* loads as no PCR amplification or weak bands were observed (samples LUNG 20, 22, 24, 30, 35, 39, 40, 45, 48, 52, and 53) in the first round of nested-PCR (mtLSUrRNA) (Supplementary Figure 3). For the real-time Msg-A/mtLSUrRNA PCR, these samples presented amplification curves with *Ct* values ranging from 35.40 to 40.00 (Table 1) and two peaks of melting curves for the two amplified genes. In addition, three samples of nasal aspirates (NA 501, 502, and 113) that were negative with the nested-PCR were positive with Msg-A/mtLSUrRNA PCR protocol observing only the melting curve of the Msg-A gene family (Supplementary Figure 4). The remaining *Pneumocystis*-negative samples were also confirmed negative by the Msg-A/mtLSUrRNA PCR. Considering the mtLSUrRNA nested-PCR as the reference method, the sensitivity, specificity, PPV and NPV values of the new real-time Msg-A/mtLSUrRNA PCR protocol were 100, 100, 100, and 100%, respectively, for the lung tissue samples group.

**TABLE 1:** Detection of *P. jirovecii* in samples by real-time Msg-A**/**mtLSUrRNA PCR compared to those amplified with the protocol developed by Rudramurthy et al. (2018) or by nested-PCR.

	Average *Ct^a^* ± SD^b^		Nested-PCR		IF
Sample	Real-time PCR Rudramurthy et al. (2018)	Real-time Msg-A/mtLSUrRNA PCR	1st Round	2nd Round	
LUNG 19	36.7 ± 0.1	26.3 ± 0.2	+	+	+
LUNG 20	>40.0 ± 0	36.4 ± 0.1	+	+	+
LUNG 21	33.8 ± 0.3	25.3 ± 0.1	+	+	+
LUNG 22	NEG	35.8 ± 0.3	−	+	−
LUNG 23	>40.0 ± 0	33.7 ± 0.3	+	+	+
LUNG 24	NEG	35.4 ± 0.3	+	+	+
LUNG 25	38.2 ± 0.1	28.9 ± 0.2	+	+	+
LUNG 26	35.6 ± 0.2	28.7 ± 0.1	+	+	+
LUNG 27	>40.0 ± 0	30.3 ± 0.1	+	+	+
LUNG 28	>40.0 ± 0	34.3 ± 0.2	+	+	+
LUNG 29	>40.0 ± 0	35.8 ± 0.3	+	+	+
LUNG 30	NEG	37.2 ± 0.1	−	+	−
LUNG 31	>40.0 ± 0	32.1 ± 0.2	+	+	+
LUNG 32	38.2 ± 0.3	29.5 ± 0.3	+	+	+
LUNG 33	>40.0 ± 0	32.6 ± 0.2	+	+	+
LUNG 34	36.6 ± 0.3	28.4 ± 0.3	+	+	+
LUNG 35	NEG	37.9 ± 0.4	−	+	−
LUNG 36	>40.0 ± 0	32.6 ± 0.4	+	+	+
LUNG 37	>40.0 ± 0	33.3 ± 0.3	+	+	+
LUNG 38	>40.0 ± 0	30.5 ± 0.2	+	+	+
LUNG 39	>40.0 ± 0	37.6 ± 0.5	+	+	−
LUNG 40	>40.0 ± 0	35.4 ± 0.3	+	+	+
LUNG 41	>40.0 ± 0	30.9 ± 0.2	+	+	+
LUNG 42	>40.0 ± 0	32.7 ± 0.3	+	+	+
LUNG 43	35.6 ± 0	26.8 ± 0.2	+	+	+
LUNG 44	>40.0 ± 0	35.1 ± 0.3	+	+	+
LUNG 45	>40.0 ± 0	36.8 ± 0.4	+	+	+
LUNG 46	37.0 ± 0.1	30.2 ± 0.3	+	+	+
LUNG 47	>40.0 ± 0	34.3 ± 0.3	+	+	+
LUNG 48	NEG	38.3 ± 0.5	+	+	+
LUNG 49	>40.0 ± 0	34.8 ± 0.4	+	+	+
LUNG 50	39.2 ± 0.2	30.0 ± 0.3	+	+	+
LUNG 51	>40.0 ± 0	33.2 ± 0.2	+	+	+
LUNG 52	>40.0 ± 0	>40.0 ± 0	+	+	−
LUNG 53	>40.0 ± 0	35.8 ± 0.4	+	+	+
LUNG 54	>40.0 ± 0	33.4 ± 0.3	+	+	+
LUNG 1650	NEG	>40.0 ± 0	+	+	+
LUNG 1783	>40.0 ± 0	38.4 ± 0.5	+	+	−
LUNG 895	>40.0 ± 0	38.8 ± 0.6	+	+	+
LUNG 2604	>40.0 ± 0	31.4 ± 0.2	+	+	+
LUNG 73B	>40.0 ± 0	32.3 ± 0.2	+	+	+
LUNG 65B	33.2 ± 0.7	25.7 ± 0.4	+	+	+
G 05v4	NEG	39.56 ± 0.3	+	+	NA
G 04v5	>40.0 ± 0	34.79 ± 0.5	+	+	NA
G 02v5	NEG	>40.0 ± 0	+	+	NA
NA 113	>40.0 ± 0	35.9 ± 0.3	−	−	NA
NA 119	>40.0 ± 0	29.7 ± 0.1	+	+	NA
NA 143	>40.0 ± 0	34.9 ± 0.5	+	+	NA
NA 501	NEG	>40.0 ± 0	−	−	NA
NA 502	NEG	>40.0 ± 0	−	−	NA
NA 150	>40.0 ± 0	27.8 ± 0.2	+	+	NA
BAL FQ773	>40.0 ± 0	34.3 ± 0.5	+	+	NA
BAL 953	>40.0 ± 0	35.9 ± 0.4	+	+	NA
BAL C965	>40.0 ± 0	33.1 ± 0.3	+	+	NA

*In the case of lung samples, immunofluorescence detection (IF) of ascus forms has also been included.*

*^a^Ct, Average threshold cycle.*

*^b^SD, Standard Deviation.*

*NA, Not applicable.*

### Comparison of Msg-A/mtLSUrRNA PCR and Rudramurthy’s PCR

To evaluate the efficiency of our Msg-A/mtLSUrRNA PCR protocol, we analyzed all *Pneumocystis*-positive samples in this study using the PCR protocol published by Rudramurthy et al. (2018). As shown in Table 1, the results differ between the two PCR protocols. Our PCR protocol presents higher sensitivity than the protocol reported by Rudramurthy et al. (2018). For *P. jirovecii*-positive samples, the *Ct* values obtained with our PCR protocol were significantly lower than those obtained by Rudramurthy‘s protocol (33.75 ± 4.0 vs. 39.18 ± 1.7, *p*-value < 0.05) (Table 1). Ten *Pneumocystis*-positive samples identified with our Msg-A/mtLSUrRNA PCR were classified negative with the PCR protocol published by Rudramurthy et al. (2018) (Supplementary Figure 5). Furthermore, because our new Msg-A/mtLSUrRNA real-time PCR protocol simultaneously amplifies two regions while the Rudramurthy et al. (2018) protocol amplifies only one, we have also compared them by amplifying the Msg region alone with our primer set Msg-A in a group of samples (LUNG 19–54). The comparison of our Msg-A primer pairs separately also obtained lower *Ct* values than those obtained with Rudramurthy’s (Msg) protocol (30.1 ± 2.36 vs. 38.01 ± 2.22, respectively, *p*-value < 0.05), thus demonstrating better optimization in the design of these new primers (Table 1).

## Discussion

In this work, we validated a laboratory-developed real-time assay for reliable detection of *P. jirovecii.* Our results showed that our Msg-A/mtLSUrRNA PCR provides sensitive data in samples containing low *P. jirovecii* loads and presents both high sensitivity and specificity. This method has several advantages: firstly, both amplification and detection steps are performed in a closed system, thus reducing the potential for contamination; secondly, it is rapid, requiring less hands-on time and allowing same-day sample analysis, with detection performed in just 3–4 h, including DNA extraction; thirdly, it is as sensitive as nested-PCR, which is considered one of the main protocols used for *P. jirovecii* detection.

Nested-PCR is based on amplifying the gene coding for *Pneumocystis* mitochondrial large-subunit (mt LSU) rRNA gene, which is present in multicopy and whose number is augmented by the number of mitochondria in fungi (Valero et al., 2016). Using multicopy genes as a target for PCR enhances its sensitivity. Robberts et al. (2007) showed that nested-PCR had the highest sensitivity to detect *P. jirovecii* in clinical samples from South African patients compared to nine other molecular assays. Although the method developed by Wakefield et al. (1990) and Wakefield (1996) became the benchmark and was taken on board for the epidemiologic investigation of *Pneumocystis* data generated by nested-PCR, it requires multiple controls and extreme care to avoid contamination. In effect, scrupulous laboratory procedures must be implemented to prevent sample contamination and ensure the absence of PCR inhibitors by applying appropriate controls.

Besides the potential problems of laboratory contamination, nested-PCR is also complicated, labor-intensive, and time-consuming (requiring 2 days). In this light, the real-time PCR method seems to be a more advantageous technique, as it is both rapid and sensitive. For the real-time Msg-A/mtLSUrRNA PCR protocol developed here, the specificity of the primers was further supported by the presence of two single fluorescence peaks in the melting curves. The slight variation recorded in the *Tm* values of each melting curve can be explained by the GC content variability within the amplified sequences in the samples (Ririe et al., 1997). Moreover, primer specificity was further corroborated by their failure to amplify DNA extracted from other pathogens typically isolated in the lung. Generally, probe-based PCR is usually used for clinical diagnosis since it is considered more specific than SYBR Green PCR. However, the use of SYBR Green is less expensive than probe synthesis, and the lower specificity of the SYBR Green affords an advantage when amplifying a heterogeneous region (Papin et al., 2004). A few mismatches within the region targeted by the probes can miss the hybridization of some sequence variants, leading to false negatives (Read et al., 2001; Varga and James, 2005). This point is of primary importance as the amplified regions used for *P. jirovecii* detection, such as the mtLSU rRNA gene or the Msg-A gene, are present in multicopy and could evolve independently. Extensive variations were observed in the Msg-A repertoire among *P. jirovecii* isolates partially generated by recombination (Kutty et al., 2008), which provide a large potential for antigenic variation and presumably facilitate evasion of immune responses in hosts (Garbe and Stringer, 1994; Wright et al., 1995). In this context, the design of primers for specific amplification of Msg-A also presents a challenge. For this reason, both Msg-A primers designed in this study contain three degenerate positions to improve the match and to amplify the greatest number of Msg-A genes showing strong specificity and amplification efficiency. On the other hand, the selection of the region for which the primer pairs were designed contained a sufficiently different percentage of GC nucleotides enabling two separate melting curves to be visualized due to their different *Tm* values or just one of them in the even that only one of the two regions was amplified. The comparison of the nested-PCR and the Msg-A/mtLSUrRNA PCR showed that three samples of nasal aspirates (NA 501, 502, and 113) were negative with the nested-PCR positive with Msg-A/mtLSUrRNA PCR protocol observing only the melting curve of the Msg-A gene family. This may be due to very low titers of *P. jirovecii*, with the detection of only the most abundant target in this sample or the existence of variability in the region of that gene where the annealing of primers takes place. Multiplex PCR reduces the number of reactions needed to test a sample for different targets and helps save time and money (Sint et al., 2012).

The comparison of Msg-A/mtLSUrRNA PCR and Rudramurthy’s PCR showed that our PCR protocol presents higher sensitivity at lower *Ct* values. In HIV patients, Rudramurthy et al. (2018) estimated that PCR amplification at a *Ct* value less than, or equal to, 25 were positive for PcP, and those that gave *Ct* values greater or equal than 26 were considered as colonized. In our study, the *Ct* values were greater than or equal to 26, which could be correlated with the *Pneumocystis* colonized status of most of our samples.

The proposed real-time Msg-A/mtLSUrRNA PCR is of value due to its increased sensitivity and specificity by improved recognition of two *P. jirovecii*-specific targets simultaneously, namely the mtLSUrRNA and the Msg-A multicopy gene family and its reduction of false positive results caused by contamination or cross-reactions. Furthermore, it provides an alternative to nested-PCR for low levels of *Pneumocystis* diagnosis, performing detection in just 3–4 h, including DNA extraction.

## Data Availability Statement

The original contributions presented in the study are included in the article/Supplementary Material, further inquiries can be directed to the corresponding author/s.

## Ethics Statement

The studies involving human participants were reviewed and approved by the Ethics Commission for Studies in Human Subjects of the University of Chile School of Medicine approved this study under protocol CEISH #092-2013. Written informed consent to participate in this study was provided by the participants’ legal guardian/next of kin.

## Author Contributions

SR-R, CP, and AM: conceptualization and writing. SR-R, CP, NP, RB, GG, VS, MG, and PB: methodology. SR-R and CP: validation and investigation and data curation. SR-R, CP, and NP: formal analysis. SR-R, CP, FM, SV, VP-B, and AM: writing—review and editing. AM: supervision. SV and AM: funding acquisition. All authors have read and agreed to the published version of the manuscript.

## Conflict of Interest

The authors declare that the research was conducted in the absence of any commercial or financial relationships that could be construed as a potential conflict of interest.

## Publisher’s Note

All claims expressed in this article are solely those of the authors and do not necessarily represent those of their affiliated organizations, or those of the publisher, the editors and the reviewers. Any product that may be evaluated in this article, or claim that may be made by its manufacturer, is not guaranteed or endorsed by the publisher.

## References

[B1] Alanio A. Desoubeaux G. Sarfati C. Hamane S. Bergeron A. Azoulay E. (2011). Real-time PCR assay-based strategy for differentiation between active *Pneumocystis jirovecii* pneumonia and colonization in immunocompromised patients. *Clin. Microbiol. Infect.* 17 1531–1537. 20946413 10.1111/j.1469-0691.2010.03400.x

[B2] Alanio A. Gits-Muselli M. Mercier-Delarue S. Dromer F. Bretagne S. (2016). Diversity of *Pneumocystis jirovecii* during infection revealed by ultra-deep pyrosequencing. *Front. Microbiol.* 7:733. 10.3389/fmicb.2016.00733 27252684 PMC4877386

[B3] Bossart S. Mühlethaler K. Garzoni C. Furrer H. (2020). Is real time PCR preferable to the direct immunofluorescence in the diagnosis of Pneumocystis jirovecii pneumonia in HIV-infected patients? *BMC Res. Notes* 13:235. 10.1186/s13104-020-05075-5 PMC7195742 32357915

[B4] Chabé M. Khalife S. Gantois N. Even G. Audebert C. (2014). An improved single-round PCR leads to rapid and highly sensitive detection of *Pneumocystis* spp. *Med. Mycol.* 52 841–846. 10.1093/mmy/myu032 24965947

[B5] Chotiprasitsakul D. Pewloungsawat P. Setthaudom C. Santanirand P. Pornsuriyasak P. (2020). Performance of real-time PCR and immunofluorescence assay for diagnosis of Pneumocystis pneumonia in real-world clinical practice. *PLoS One* 15:e0244023. 10.1371/journal.pone.0244023 33347478 PMC7751978

[B6] Dellière S. Gits-Muselli M. White P. L. Mengoli C. Bretagne S. Alanio A. (2019). Quantification of *Pneumocystis jirovecii*: cross-platform comparison of one qPCR assay with leading platforms and six master mixes. *J. Fungi* 6:9. 10.3390/jof6010009 PMC7151141 31888050

[B7] Dunaiski C. M. Janssen L. Erzinger H. Pieper M. Damaschek S. Schildgen O. (2018). Inter-specimen imbalance of mitochondrial gene copy numbers predicts clustering of *Pneumocystis jirovecii* isolates in distinct subgroups. *J. Fungi* 4:84. 10.3390/jof4030084 29996561 PMC6162491

[B8] Esteves F. Gaspar J. De Sousa B. Antunes F. Mansinho K. Matos O. (2011). Clinical relevance of multiple single-nucleotide polymorphisms in *Pneumocystis jirovecii* Pneumonia: development of a multiplex PCR-single-base-extension methodology. *J. Clin. microbiol.* 49 1810–1815. 21389160 10.1128/JCM.02303-10 PMC3122690

[B9] Fauchier T. Hasseine L. Gari-Toussaint M. Casanova V. Marty P. M. Pomares C. (2016). Detection of *Pneumocystis jirovecii* by quantitative PCR to differentiate colonization and pneumonia in immunocompromised HIV-positive and HIV-negative patients. *J. Clin. Microbiol.* 54 1487–1495. 10.1128/JCM.03174-15 27008872 PMC4879311

[B10] Fraczek M. G. Ahmad S. Richardson M. Kirwan M. Bowyer P. Denning D. W. (2019). Detection of *Pneumocystis jirovecii* by quantitative real-time PCR in oral rinses from Pneumocystis pneumonia asymptomatic human immunodeficiency virus patients. *J. Med. Mycol.* 29 107–111. 10.1016/j.mycmed.2019.04.001 31047784

[B11] Garbe T. R. Stringer J. R. (1994). Molecular characterization of clustered variants of genes encoding major surface antigens of human *Pneumocystis carinii*. *Infect Immun.* 62 3092–3101. 10.1128/iai.62.8.3092-3101 7518806 PMC302932

[B12] Gits-Muselli M. White P. L. Mengoli C. Chen S. Crowley B. Dingemans G. (2020). The Fungal PCR Initiative’s evaluation of in-house and commercial *Pneumocystis jirovecii* qPCR assays: toward a standard for a diagnostics assay. *Med. Mycol.* 58 779–788. 10.1093/mmy/myz115 31758173

[B13] Kutty G. Maldarelli F. Achaz G. Kovacs J. A. (2008). Variation in the major surface glycoprotein genes in *Pneumocystis jirovecii*. *J. Infect. Dis.* 198 741–749. 18627244 10.1086/590433 PMC2561252

[B14] Linssen C. F. M. Jacobs J. A. Beckers P. Templeton K. E. Bakkers J. Kuijper E. J. (2006). Inter-laboratory comparison of three different real-time PCR assays for the detection of *Pneumocystis jirovecii* in bronchoalveolar lavage fluid samples. *J. Med. Microbiol.* 55 1229–1235. 10.1099/jmm.0.46552-0 16914653

[B15] Ma L. Chen Z. Huang da W. Kutty G. Ishihara M. Wang H. (2016). Genome analysis of three *Pneumocystis* species reveals adaptation mechanisms to life exclusively in mammalian hosts. *Nat. Commun.* 7:10740. 10.1038/ncomms10740 PMC4764891 26899007

[B16] Montesinos I. Delforge M. L. Ajjaham F. Brancart F. Hites M. Jacobs F. (2017). Evaluation of a new commercial real-time PCR assay for diagnosis of *Pneumocystis jirovecii* pneumonia and identification of dihydropteroate synthase (DHPS) mutations. *Diagn. Microbiol. Infect. Dis.* 87 32–36. 10.1016/j.diagmicrobio.2016.10.005 27789058

[B17] Morilla R. González-Magaña A. Friaza V. de Armas Y. Medrano F. J. Calderón E. J. (2019). Genetic polymorphisms of superoxide dismutase locus of *Pneumocystis jirovecii* in Spanish population. *Front. Public Health* 7:292. 10.3389/fpubh.2019.00292 31681723 PMC6803434

[B18] Morris A. Wei K. Afshar K. Huang L. (2008). Epidemiology and clinical significance of *Pneumocystis* colonization. *J. Infect. Dis.* 97 10–17. 10.1086/523814 18171279

[B19] Özkoç S. Köker M. Önder M. Delibaş S. B. (2016). Prevalence of *Pneumocystis jirovecii* colonization in autopsy cases in Turkey. *J. Med. Microbiol.* 65 1152–1157. 10.1099/jmm.0.000337 27542715

[B20] Papin J. F. Vahrson W. Dittmer D. P. (2004). SYBR green-based real-time quantitative PCR assay for detection of West Nile Virus circumvents false-negative results due to strain variability. *J. Clin. Microbiol.* 42 1511–1518. 10.1128/JCM.42.4.1511-1518.2004 15070997 PMC387603

[B21] Ponce C. A. Gallo M. Bustamante R. Vargas S. L. (2010). *Pneumocystis* colonization is highly prevalent in the autopsied lungs of the general population. *Clin. Infect. Dis.* 50 347–353. 20047487 10.1086/649868

[B22] Read S. J. Mitchell J. J. Fink C. G. (2001). LightCycler multiplex PCR for the laboratory diagnosis of common viral infections of the central nervous system. *J. Clin. Microbiol.* 39 3056–3059. 10.1128/JCM.39.9.3056-3059.2001 11526128 PMC88296

[B23] Ririe K. Rasmussen R. P. Wittwer C. T. (1997). Product differentiation by analysis of DNA melting curves during the polymerase chain reaction. *Anal. Biochem.* 245 154–160. 9056205 10.1006/abio.1996.9916

[B24] Robberts F. J. Liebowitz L. D. Chalkley L. J. (2007). Polymerase chain reaction detection of *Pneumocystis jirovecii*: evaluation of 9 assays. *Diagn. Microbiol. Infect. Dis.* 58 385–392. 10.1016/j.diagmicrobio.2007.02.014 17689766

[B25] Robert-Gangneux F. Belaz S. Revest M. Tattevin P. Jouneau S. Decaux O. (2014). Diagnosis of *Pneumocystis jirovecii* pneumonia in immunocompromised patients by real-time PCR: a 4-year prospective study. *J. Clin. Microbiol.* 52 3370–3376. 10.1128/JCM.01480-14 25009050 PMC4313164

[B26] Rudramurthy S. M. Sharma M. Sharma M. Rawat P. Ghosh A. Venkatesan L. (2018). Reliable differentiation of *Pneumocystis* pneumonia from *Pneumocystis* colonisation by quantification of major surface glycoprotein gene using real-time polymerase chain reaction. *Mycoses* 61 96–103. 28945326 10.1111/myc.12708

[B27] Ruiz-Ruiz S. Ponce C. A. Pesantes N. Bustamante R. Gatti G. San Martin V. (2019). New DNA extraction method for the detection of *Pneumocystis* in lung tissue samples of colonized individuals. *OBM Genet.* 3:11. 10.21926/obm.genet.1901066

[B28] Sarasombath P. T. Thongpiya J. Chulanetra M. Wijit S. Chinabut P. Ongrotchanakun J. (2021). Quantitative PCR to discriminate between pneumocystis pneumonia and colonization in HIV and Non-HIV immunocompromised patients. *Front. Microbiol.* 12:729193. 10.3389/fmicb.2021.729193 PMC8564139 34745031

[B29] Sint D. Raso L. Traugott M. (2012). Advances in multiplex PCR: balancing primer efficiencies and improving detection success. *Methods Ecol. Evol.* 3 898–905. 10.1111/j.2041-210X.2012.00215.x 23549328 PMC3573865

[B30] Thompson J. D. Gibson T. J. Higgins D. G. (2002). Multiple sequence alignment using ClustalW and ClustalX*. *Curr. Protoc. Bioinformatics* 10.1002/0471250953.bi0203s00 18792934

[B31] Valero C. Buitrago M. J. Gits-Muselli M. Benazra M. Sturny-Leclère A. Hamane S. (2016). Copy number variation of mitochondrial DNA genes in *Pneumocystis jirovecii* according to the fungal load in BAL specimens. *Front. Microbiol.* 12:1413. 10.3389/fmicb.2016.01413 PMC5018473 27672381

[B32] Varga A. James D. (2005). Detection and differentiation of Plum pox virus using real-time multiplex PCR with SYBR Green and melting curve analysis: a rapid method for strain typing. *J. Virol. Methods* 123 213–220. 15620404 10.1016/j.jviromet.2004.10.005

[B33] Vargas S. L. Ponce C. Bustamante R. Calderón E. Nevez G. De Armas Y. (2017). Importance of tissue sampling, laboratory methods, and patient characteristics for detection of *Pneumocystis* in autopsied lungs of non-immunosuppressed individuals. *Eur. J. Clin. Microbiol. Infect. Dis.* 36 1711–1716. 10.1007/s10096-017-3006-8 28584896 PMC5602097

[B34] Wakefield A. E. (1996). DNA sequences identical to *Pneumocystis carinii* f. sp. *carinii* and *Pneumocystis carinii* f. sp. *hominis* in samples of air spora. *J. Clin. Microbiol.* 34 1754–1759. 10.1128/JCM.34.7.1754-1759.1996 8784583 PMC229108

[B35] Wakefield A. E. Pixley F. J. Banerji S. Sinclair K. Miller R. F. Moxon E. R. (1990). Detection of *Pneumocystis carinii* with DNA amplification. *Lancet* 336 451–453. 10.1016/0140-6736(90)92008-6 1974987

[B36] Wright T. W. Bissoondial T. Y. Haidaris C. G. Gigliotti F. Haidaris P. J. (1995). Isoform diversity and tandem duplication of the glycoprotein A gene in ferret *Pneumocystis carinii*. *DNA Res.* 2 77–88. 10.1093/dnares/2.2.77 7584051

[B37] Yang S. L. Wen Y. H. Wu Y. S. Wang M. C. Chang P. Y. Yang S. (2020). Diagnosis of pneumocystis pneumonia by real-time PCR in patients with various underlying diseases. *J. Microbiol. Immunol. Infect.* 53 785–790. 31635929 10.1016/j.jmii.2019.08.012

