# Correction: A real-time PCR assay for detection of low *Pneumocystis jirovecii* levels

**DOI:** 10.3389/fmicb.2026.1861741

**Published:** 2026-05-12

**Authors:** Susana Ruiz-Ruiz, Carolina A. Ponce, Nicole Pesantes, Rebeca Bustamante, Gianna Gatti, Viviana San Martin, Mireya Gutierrez, Pamela Bórquez, Sergio L. Vargas, Fabien Magne, Enrique J. Calderón, Vicente Pérez-Brocal, Andrés Moya

**Affiliations:** 1 Fundación para el Fomento de la Investigación Sanitaria y Biomédica de la Comunitat Valenciana (FISABIO)-Salud Pública, València, Spain; 2 Centro de Investigación Biomédica en Red en Epidemiología y Salud Pública (CIBEResp), Instituto de Salud Carlos III, Madrid, Spain; 3 Programa de Microbiología y Micología, Facultad de Medicina, Instituto de Ciencias Biomédicas, Universidad de Chile, Santiago, Chile; 4 Servicio Médico Legal, Santiago, Chile; 5 Hospital Universitario Virgen del Rocío, Instituto de Biomedicina de Sevilla, Consejo Superior de Investigaciones Científicas (CSIC), and Universidad de Sevilla, Seville, Spain; 6 Instituto de Biología Integrativa de Sistemas (I2Sysbio), Universitat de València and Consejo Superior de Investigaciones Científicas (CSIC), València, Spain

**Keywords:** real-time PCR, low *P. jirovecii* loads, major surface glycoproteins, mitochondrial large subunit rRNA, simultaneous detection

In the published article, there was a mistake in the **Funding** statement. The **Funding** statement for the Spanish Ministry of Science and Innovation was displayed as (PID2019-105969GB-I00). The correct statement is “Spanish Ministry of Science and Innovation PID2019-105969GB-I00 funded by MICIU/AEI/10.13039/501100011033′'.

The correct **Funding** Section is:

“The author(s) declared that financial support was received for this work and/or its publication. This research was funded by the ERANet LAC (ELAC2014/HID-0254), the National Fund for Science and Technology (Fondecyt, Chile) (1140412), the Spanish Ministry of Science and Innovation PID2019-105969GB-I00 funded by MICIU/AEI /10.13039/501100011033, Generalitat Valenciana (Spain) (Prometeo/2018/A/133), and co-financed by the European Regional Development Fund (ERDF, EU)”.

The original version of this article has been updated.

