# Supplementary material for: A Real-Time PCR Assay for Detection of Low Pneumocystis jirovecii Levels
Source: Front Microbiol. 2022 Jan 11;12:787554. doi: 10.3389/fmicb.2021.787554 (PMC8787145; doi:10.3389/fmicb.2021.787554)
Supplement: Supplementary file 1 [file Data_Sheet_1.PDF]

Table S1. Detection of *Pneumocystis jirovecii* in lung samples (LUNG 19-54) by real-time Msg-A PCR compared to those amplified with the developed by Rudramurthy et al. (2018).

Sample	Average *Ct*[1] ± SD[2]	
	Rudramurthy et al. (2018)	Real-time Msg-A PCR
LUNG 19	36.7 ± 0.1	27.8 ± 01
LUNG 20	>40.0 ± 0	37.2 ± 0.1
LUNG 21	33.8 ± 0.3	26.1 ± 0.1
LUNG 22	NEG	37.2 ± 0.3
LUNG 23	>40.0 ± 0	34.9 ± 0.2
LUNG 24	NEG	36.9 ± 0.3
LUNG 25	38.2 ± 0.1	29.7 ± 0.2
LUNG 26	35.6 ± 0.2	29.6 ± 0.1
LUNG 27	>40.0 ± 0	31.7 ± 0.1
LUNG 28	>40.0 ± 0	34.3 ± 0.2
LUNG 29	>40.0 ± 0	35.8 ± 0.3
LUNG 30	NEG	37.9 ± 0.1
LUNG 31	>40.0 ± 0	33.4 ± 0.1
LUNG 32	38.2 ± 0.3	30.0 ± 0.1
LUNG 33	>40.0 ± 0	33.7 ± 0.3
LUNG 34	36.6 ± 0.3	29.6 ± 0.2
LUNG 35	NEG	38.6 ± 0.5
LUNG 36	>40.0 ± 0	33.5 ± 0.8
LUNG 37	>40.0 ± 0	34.2 ± 0.7
LUNG 38	>40.0 ± 0	31.5 ± 0.2
LUNG 39	>40.0 ± 0	38.8 ± 1.0
LUNG 40	>40.0 ± 0	36.6 ± 0.1
LUNG 41	>40.0 ± 0	31.9 ± 0.1
LUNG 42	>40.0 ± 0	32.7 ± 0.1
LUNG 43	35.6 ± 0	28.0 ± 0.1
LUNG 44	>40.0 ± 0	35.4 ± 0.1
LUNG 45	>40.0 ± 0	37.5 ± 0.3
LUNG 46	37.0 ± 0.1	31.2 ± 0.8
LUNG 47	>40.0 ± 0	34.8 ± 0.9
LUNG 48	NEG	39.6 ± 0.6
LUNG 49	>40.0 ± 0	35.6 ± 1.4
LUNG 50	39.2 ± 0.2	30.6 ± 0.1
LUNG 51	>40.0 ± 0	34.4 ± 0.6
LUNG 52	>40.0 ± 0	40.0 ± 0.0
LUNG 53	>40.0 ± 0	36.3 ± 0.2
LUNG 54	>40.0 ± 0	34.1 ± 0.3

[1] *Ct* = Average threshold cycle

[2] SD = Standard Deviation

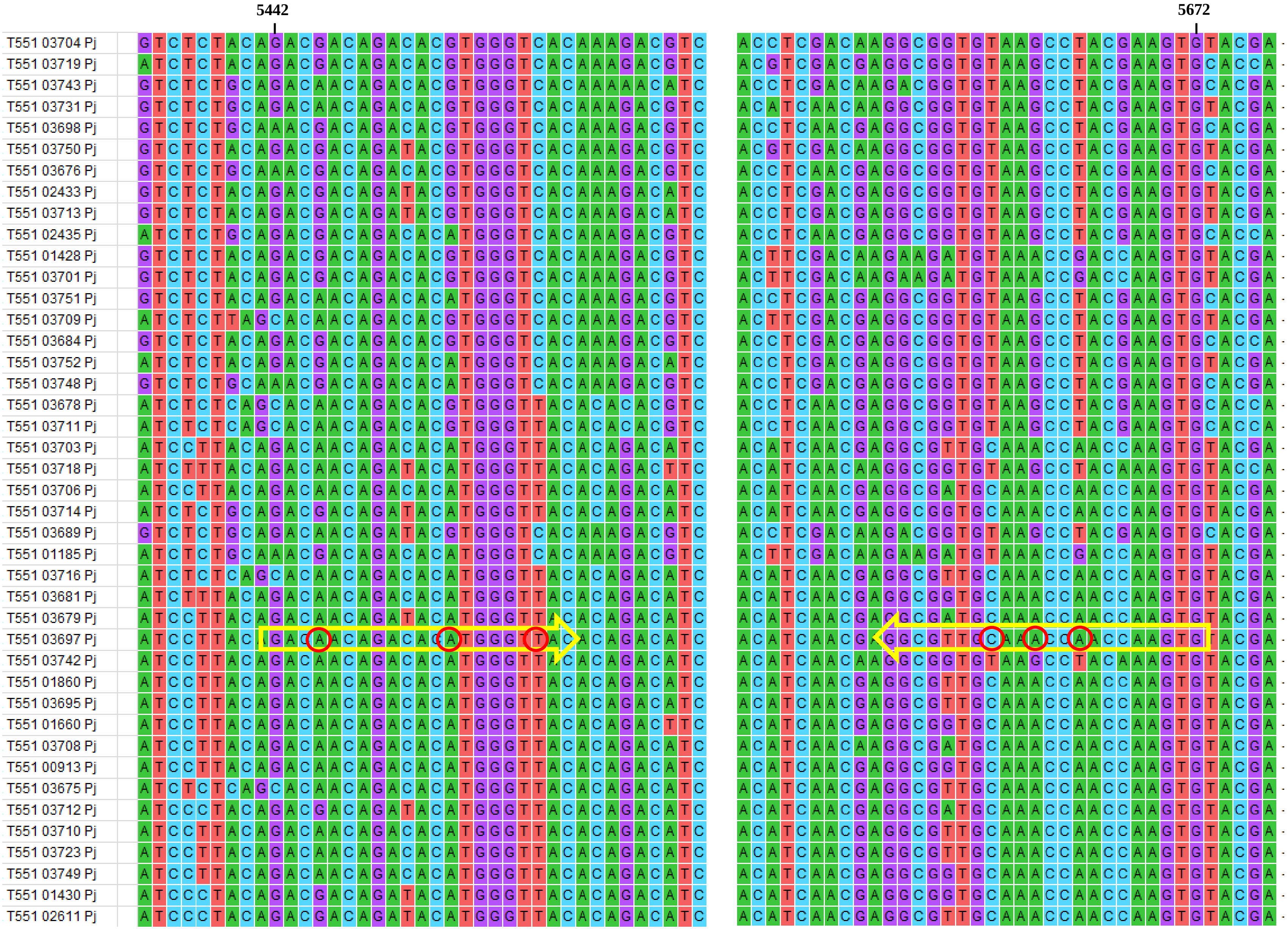

Figure S1. Alignment of Msg DNA sequences. Part of the multiple alignment of all Msg DNA sequences available retrieved from the original publication Ma et al. (2016) of the 3´ terminal region. The nucleotide sequence of the primer pair Msg-AF - Msg-AR are in yellow and in red are highlighted degenerate positions.

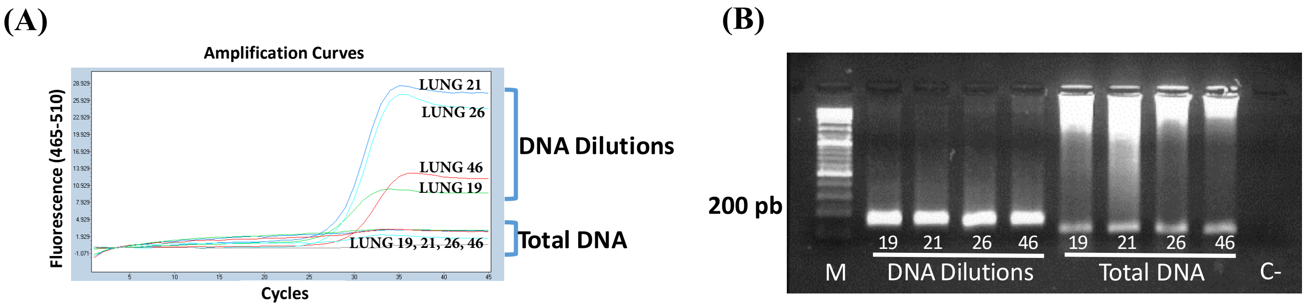

Figure S2. Amplification differences observed by real-time Msg-A/mtLSUrRNA PCR from DNA dilutions (10ng/µl) or total DNA of samples LUNG 19, 21, 26 and 46. (A) Amplification curves obtained from samples. (B) PCR amplification products obtained in 2% agarose gel electrophoresis. Lane 1: 1 kb plus ladder DNA marker.

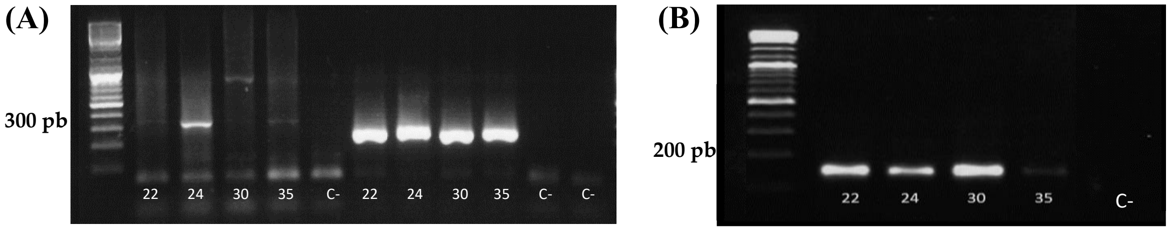

Figure S3. Electrophoretic migration in a 2% agarose gel of the amplification products obtained from DNA extracts of samples LUNG 22, 24, 30, and 35. (A) Amplification products obtained by two rounds of nested-PCR. (B) Amplification products obtained by real-time Msg-A/mtLSUrRNA PCR. Lane 1: 1 kb plus ladder DNA marker.

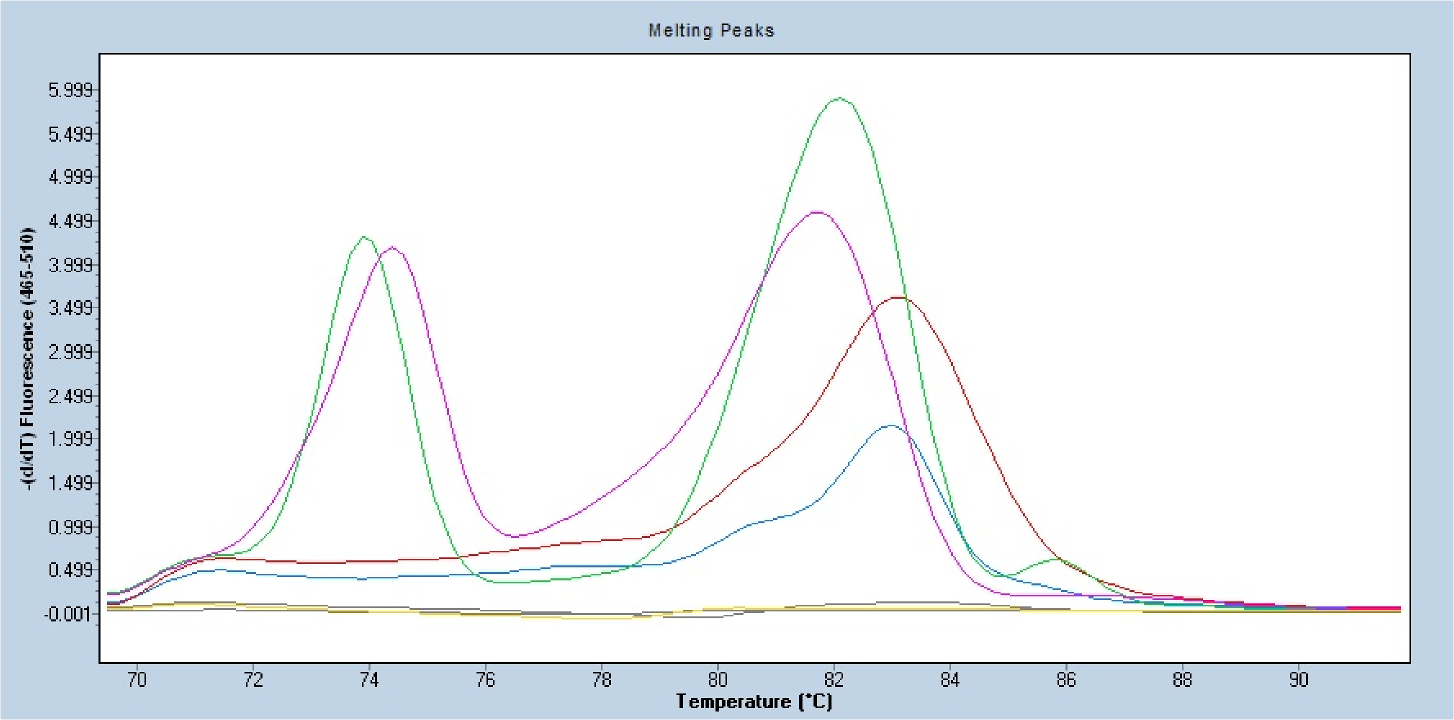

Figure S4. First derivative of individual melting curves of the samples NA 501, NA 502, LUNG 21 and LUNG 25. Two peaks of melting curves for the two amplified genes were observed for the samples LUNG 21 and LUNG 25, while in NA 501 and NA 502 only the melting curve of the Msg-A gene family. No melting curves profile were detected in negative controls.

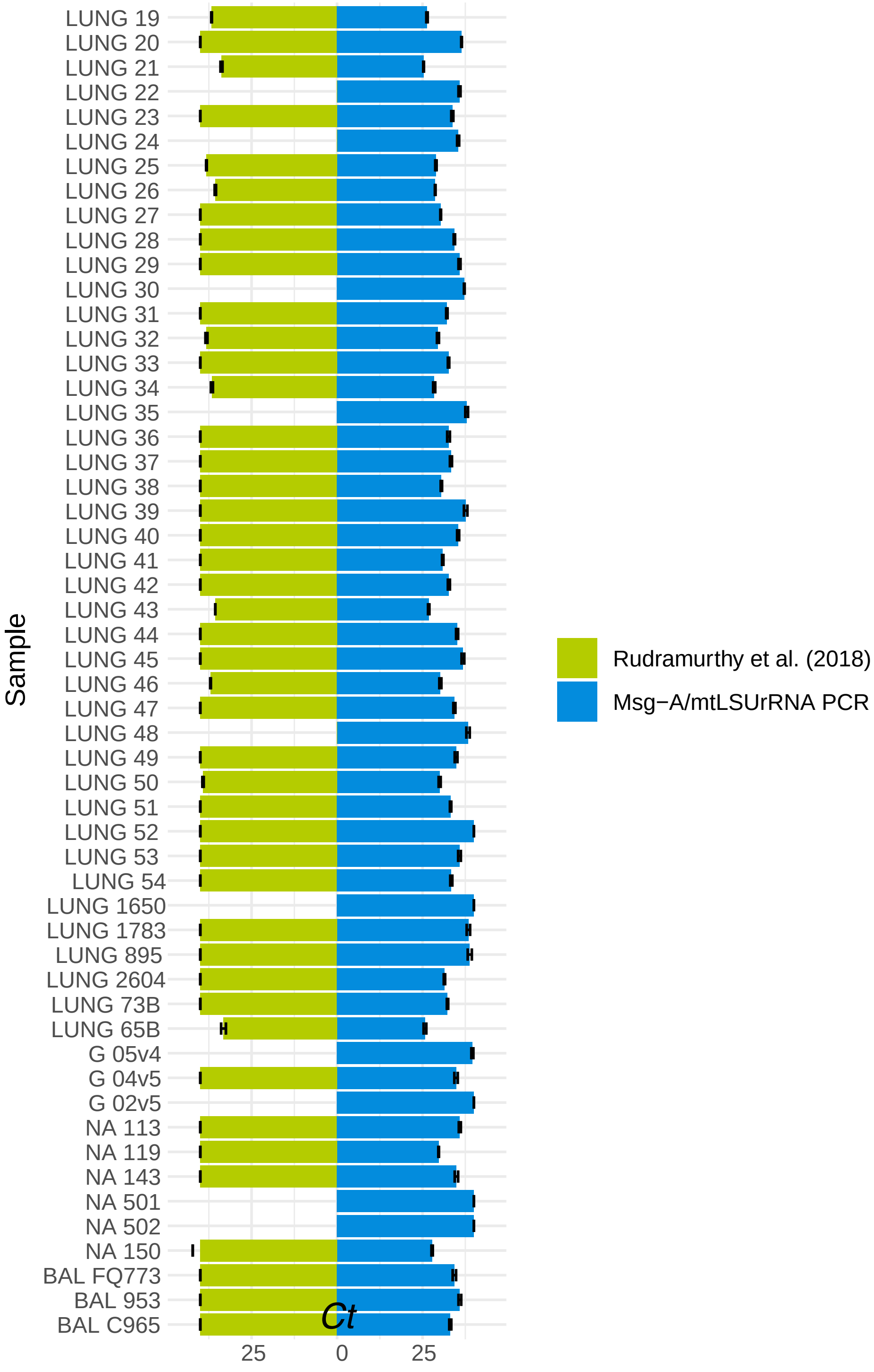

Figure S5. Box plot comparing the *Ct* values (with Standard Deviations in black) obtained by the Msg-A/mtLSUrRNA and by Rudramurthy et al. (2018) PCR.
